# Functional Groups Determine Biochar Properties (pH and EC) as Studied by Two-Dimensional ^13^C NMR Correlation Spectroscopy

**DOI:** 10.1371/journal.pone.0065949

**Published:** 2013-06-19

**Authors:** Xiaoming Li, Qirong Shen, Dongqing Zhang, Xinlan Mei, Wei Ran, Yangchun Xu, Guanghui Yu

**Affiliations:** 1 Agricultural Ministry Key Lab of Plant Nutrition and Fertilization in Low-Middle Reaches of the Yangtze River, Nanjing, PR China; 2 College of Resources and Environmental Sciences, Nanjing Agricultural University, Nanjing, PR China; 3 Baoshan Environmental Protection Bureau, Shanghai, PR China; National Research Council of Italy, Italy

## Abstract

While the properties of biochar are closely related to its functional groups, it is unclear under what conditions biochar develops its properties. In this study, two-dimensional (2D) ^13^C nuclear magnetic resonance (NMR) correlation spectroscopy was for the first time applied to investigate the development of functional groups and establish their relationship with biochar properties. The results showed that the agricultural biomass carbonized to biochars was a dehydroxylation/dehydrogenation and aromatization process, mainly involving the cleavage of O-alkylated carbons and anomeric O-C-O carbons in addition to the production of fused-ring aromatic structures and aromatic C-O groups. With increasing charring temperature, the mass cleavage of O-alkylated groups and anomeric O-C-O carbons occurred prior to the production of fused-ring aromatic structures. The regression analysis between functional groups and biochar properties (pH and electrical conductivity) further demonstrated that the pH and electrical conductivity of rice straw derived biochars were mainly determined by fused-ring aromatic structures and anomeric O-C-O carbons, but the pH of rice bran derived biochars was determined by both fused-ring aromatic structures and aliphatic O-alkylated (HCOH) carbons. In summary, this work suggests a novel tool for characterising the development of functional groups in biochars.

## Introduction

Agricultural biomass is one of the most abundant renewable resources on Earth. The thermal transformation of agricultural biomass into biochar in an oxygen-depleted atmosphere has been extensively studied, because of concerns over enhancement of soil fertility and mitigation of climate change by long-term carbon sequestration [1], [2]. The main functional groups of biochar are aromatic and heterocyclic carbons, which are believed to be stable in soil due to their chemical recalcitrance [3], [4]. Therefore, the characterization of functional groups in biochar is critical to understanding the reaction mechanisms of the charring processes. Furthermore, acquiring clear knowledge of biochar functional groups is important for the beneficial use of biochar products.

The properties of biochar greatly depend on the production conditions (i.e., charring temperature) and the biomass type used to produce biochar [5], [6], [7]. In general, biochar produced below 400°C had a low pH, low electrical conductivity (EC), and small surface area [5]. The properties of biochar were closely related to its functional groups in the charring temperatures range of 100∼600°C [7]. However, it is unclear under what conditions biochar develops its properties [5], [8].

Solid-state ^13^C nuclear magnetic resonance (NMR) spectroscopy has been frequently employed to characterize the functional groups of biochar [9], [10], [11], [12]. However, the individual spectral features of ^13^C NMR often overlap because of the extreme heterogeneity of biochar. Therefore, it is difficult to establish the relationship between biochar properties and functional groups. Recently, we have applied two-dimensional (2D) correlation spectroscopy to environmental sciences for the purpose of unrevealing the overlapped peaks [13], [14], [15]. The main advantages of 2D correlation spectroscopy are as follows: 1) providing a better resolution of significant peaks; and 2) the ability to allow for probe the specific sequencing of spectral intensity changes through asynchronous analysis [13], [14], [15]. By applying 2D correlation spectroscopy, it is expected that the best possible biochar may be “designed” for a given application, based on assessments of the dynamic functional groups of biochar and their relationship with the corresponding properties.

The objectives of the present study were to 1) investigate the development of functional groups in biomass-derived biochar by applying 2D correlation spectroscopy and 2) establish the relationship between functional groups and biochar properties (i.e., pH and EC). For these purposes, two types of agricultural biomass, i.e. rice straw and rice bran, were selected to produce biochars.

## Materials and Methods

### Biochar Preparation

Rice straw and rice bran were collected from Yixing city, Jiangsu Province, China. No specific field permits were required for this study. The land accessed is not privately owned or protected. No protected species were sampled. Biochars were produced via pyrolyzing biomass in a furnace (SXL-1200, Shanghai Daheng Optics and Fine Mechanics Co., Ltd., Shanghai, China) at various temperatures under oxygen-limited conditions. The furnace could provide a constant temperature environment of 100–800°C at an averagely 10°C min^−1^ rate of temperature increase. All biochar samples were then examined for their physical and chemical characteristics.

### Solid-state ^13^C NMR Spectroscopy

Solid-state ^13^C NMR spectroscopy was conducted on a Bruker AV-400, equipped with a 4-mm wide-bore MAS probe [13]. NMR spectra were obtained by applying the following parameters: rotor spin rate of 13000 Hz, 1 s recycle time, 1 ms contact time, 20 ms acquisition time, and 4000 scans. Samples were packed in 4-mm zirconia rotors with Kel-F caps. The pulse sequence was applied with a ^1^H ramp to account for non-homogeneity of the Hartmann-Hahn condition at high spin rotor rates. Chemical shifts were calibrated with adamantine. It should be noted that solid-state ^13^C NMR spectroscopy is only a quanlitative rather than quantitative method [16].

### Analysis of 2D Correlation Spectroscopy

The 2D correlation spectra were produced according to the method of Noda and Ozaki [17]. In the present study, the charring temperature was applied as an external perturbation, and a set of temperature-dependent NMR spectra was obtained. Let us consider the analytical spectrum *I(x, t)*. The variable *x* is the index variable representing the NMR spectra induced by the perturbation variable *t*. We intentionally use *x* instead of the general notation used in conventional 2D correlation equations based on the spectral index *v*. The analytical spectrum *I(x, t)* at *m* with evenly spaced points in *t* (between *T_min_* and *T_max_*) can be represented as
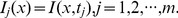
(1)A set of dynamic spectra is given by:
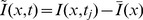
(2)where 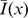
denotes the reference spectrum, which is typically the average spectrum and is expressed as 
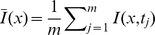
. The synchronous correlation intensity can be directly calculated from the following dynamic spectra:
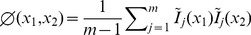
(3)Asynchronous correlation can be obtained by:
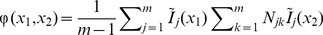
(4)The term 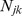 corresponds to the *j* ^th^ column and the *k* ^th^ raw element of the discrete Hilbert-Noda transformation matrix, which is defined as
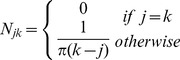
(5)The intensity of a synchronous correlation spectrum (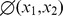) represents simultaneous changes in two spectral intensities measured at *x_1_* and *x_2_* during the interval between *T_min_* and *T_max_*. In contrast, an asynchronous correlation spectrum (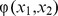) includes out-of-phase or sequential changes in spectral intensities measured at *x_1_* and *x_2_*.

Prior to 2D analysis, the NMR spectra were normalized by summing the absorbance from 200-0 ppm and multiplying by 1000. Subsequently, 2D correlation spectroscopy was conducted using 2Dshige software (Kwansei Gakuin University, Japan).

### Chemical Characterization Assay

Biochar (<0.25 mm) was soaked with deionized water at a 1∶5 solid/water ratio for 24 h with agitation. The slurry was then measured for pH using a pH electrode and for electrical conductivity (EC) using a conductivity meter (LF91, German). Elemental composition (CHNSO) was determined by dry combustion using a Perkin–Elmer2400 CHNS elemental analyzer. The O content was calculated by O% = 100-C%-H%-N%-S%.

### Statistical Analysis

Statistical analysis was performed using the software SPSS version 16.0 for Windows (SPSS, Chicago, IL). Pearson's correlation coefficient (*R*) was used to evaluate the linear correlation between biochar properties (pH and EC) and functional groups. Pearson's coefficient is always between −1 and +1, where −1 denotes a perfect negative correlation, +1 denotes a perfect positive correlation, and 0 denotes the absence of a relationship. The correlations were considered to be statistically significant at a 95% confidence interval (*p*<0.05).

## Results and Discussion

### Properties of Biochars at Various Charring Temperatures

With increasing charring temperature, yields declined rapidly at 300°C and remained relatively stable above 600°C for both biochars (Table 1). Final yields of biochars were approximately 10–14% for both biochars. The C content increased, whereas the H, N, S and O contents decreased with increasing charring temperature. All of the atomic ratios [i.e., H/C, O/C and (O+N)/C] sharply decreased, suggesting that with increasing charring temperature, the relative degree of aromaticity (H/C ratio) and polarity [O/C and (O+N)/C ratios] markedly decreased, which could be attributable to the development of functional groups and will be discussed in the next sections.

**Table 1:** Yields, elemental composition, atomic ratios, pH and EC of rice straw and rice bran derived biochars prepared at various charring temperatures.^a^

material	charring temperature (°C)	yield/%	elemental composition (%)					atomic ratios			pH	EC (mS/cm)
			C	H	N	S	O	H/C	O/C	(O+N)/C		
Rice straw	100	97.2±0.0	39.13	4.58	3.72	3.41	49.16	1.40	0.94	1.02	5.25±0.04	4.09±0.10
	200	77.1±0.9	42.02	3.34	3.04	2.15	49.45	0.95	0.88	0.94	5.93±0.01	5.64±0.02
	300	30.1±1.8	49.68	2.17	2.07	1.68	44.40	0.52	0.67	0.70	7.16±0.05	5.57±0.02
	400	26.0±1.7	61.24	1.51	1.07	1.02	35.16	0.30	0.43	0.44	8.41±0.11	6.22±0.12
	500	19.7±2.3	69.78	0.99	0.98	0.89	27.36	0.17	0.29	0.31	10.1+0.19	6.59±0.01
	600	13.5±2.2	77.24	0.87	0.81	0.72	20.36	0.14	0.20	0.21	10.6±0.05	7.31±0.05
	700	12.8±0.6	86.99	0.85	0.88	0.75	10.53	0.12	0.09	0.10	10.3±0.03	7.51±0.07
	800	10.7±0.8	88.12	0.83	0.84	0.81	9.40	0.11	0.08	0.09	10.5±0.02	7.72±0.02
	100	97.4±0.2	42.39	4.21	3.24	2.17	47.99	1.19	0.85	0.91	5.48±0.01	2.84±0.02
Rice bran	200	83.9±0.0	46.53	2.47	2.15	1.84	47.01	0.64	0.76	0.79	6.16±0.01	2.56±0.02
	300	31.1±1.9	50.68	2.14	1.58	1.51	44.09	0.51	0.65	0.68	6.89±0.02	1.12±0.01
	400	24.5±1.1	64.05	1.16	1.34	0.92	32.53	0.22	0.38	0.40	7.43±0.02	2.20±0.03
	500	18.5±2.4	71.72	1.01	1.03	0.84	25.40	0.17	0.27	0.28	8.95±0.02	3.24±0.071
	600	18.0±1.9	77.58	0.79	0.72	0.79	20.12	0.12	0.19	0.20	10.04±0.09	3.67±0.03
	700	15.4±2.1	82.03	0.84	0.74	0.81	15.52	0.12	0.14	0.15	10.28±0.08	3.74±0.03
	800	14.4±3.1	83.14	0.84	0.77	0.80	14.45	0.12	0.13	0.14	10.92±0.06	3.44±0.03

a **All chemical analyses were carried out in triplicate.**

**Figure 1:**
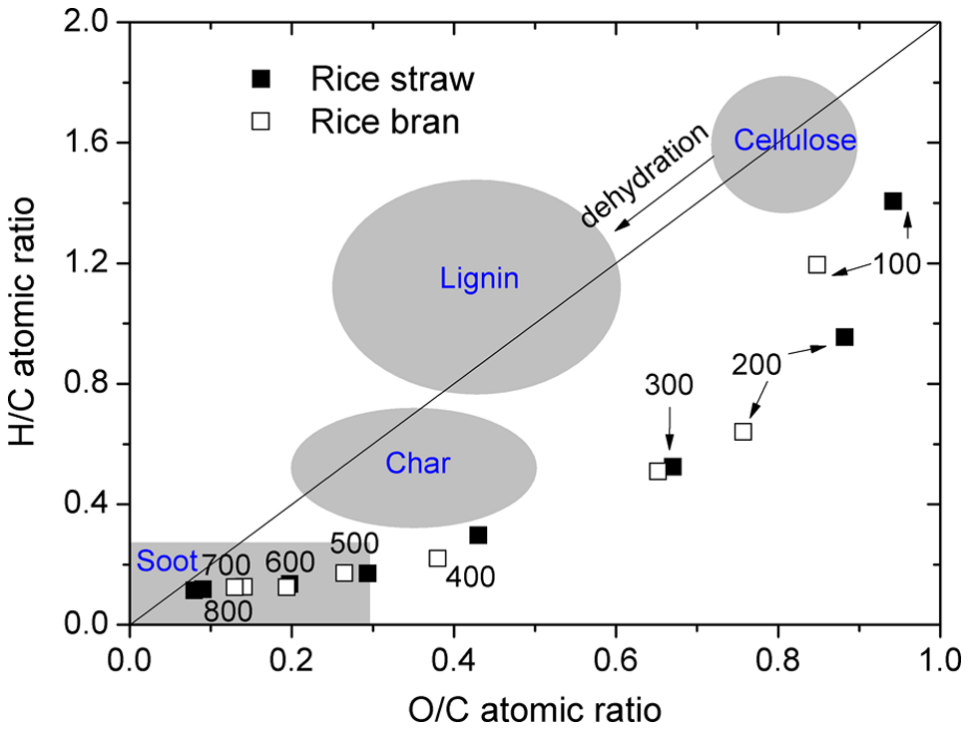
Van Krevelen plot of elemental ratios for rice straw (▪) and sawdust (□) derived biochars prepared at various charring temperatures. Thick line represents the direction for dehydration reactions and grey shadings highlight approximate elemental ratios of unaltered biomacromolecules (cellulose and lignin) and black carbon materials (char and soot) following Hammes et al. [16].

The typical van Krevelen plot showed that the progressive decrease in the H/C and O/C atomic ratios with temperature followed the trajectory associated with dehydration reactions (Figure 1). The most dramatic loss of H and O occurred in the range of 300–400°C for both biochars. Moreover, the biochars produced at 400 and 500°C were expected to have characteristics of chars, which is consistent with biochar yields (Table 1). Above 600°C, the biochars may be considered to be soot [18]. These trends in elemental composition and atomic ratios with the various charring temperatures were consistent with the previous investigations [19], [20], [21].

The charring of rice straw and rice bran increased their pH (Table 1). The pH of charred rice straw and sawdust increased from acidic (approximately 5) to alkaline (approximately 10) for both biochars. However, the charring of rice straw increased its EC, but the charring of rice bran decreased its EC before 300°C and then increased its EC (except for 800°C). The EC of charred rice straw gradually increased from 4.09±0.10 at 100°C to 7.72±0.02 at 800°C. However, the development of EC for charred rice bran fluctuated. Specifically, the EC decreased from 2.84±0.02 at 100°C to 1.12±0.01 at 300°C and then increased to 3.44±0.03 at 800°C. The differences between charred rice straw and rice bran should be attributed to the nature of plant biomass. These results were similar to those of Lehmann [5], supporting that the properties of biochar were greatly dependent on the production procedure and raw material.

### Development of Functional Groups during the Charring Process

**Figure 2:**
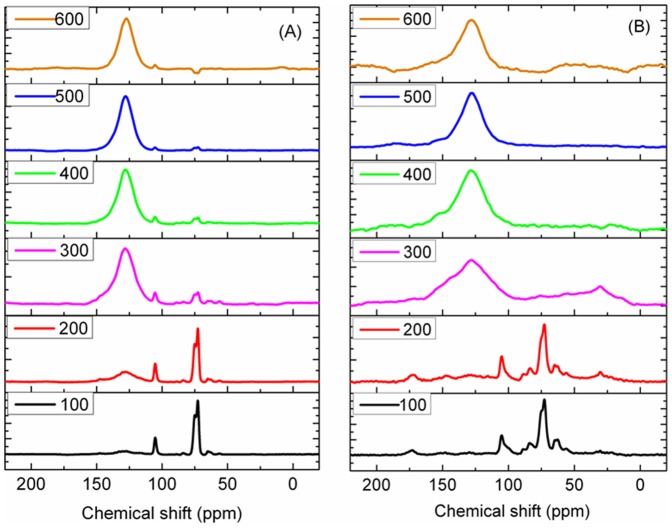
Solid-state ^13^C NMR spectra of rice straw (A) and rice bran (B) derived biochars prepared at various charring temperatures.

One-dimensional NMR spectra of rice straw and rice bran derived biochars (Figures 2-a and 2-b) showed a two-phase characteristic. Note that no NMR signal was detected for biochars at 700 and 800°C, which is most likely attributable to the high EC that affected the alignment of aromatic sheets [22], an increase in conductivity of the sample, or the occurrence of a larger number of delocalized π electrons around the ^13^C nuclei arising from the growth of the aromatic planes of the chars at higher temperature [23]. The transition charring temperature occurred at 300°C. In the first phase, aliphatic O-alkylated (HCOH) (73 ppm) carbons were predominant in biochars. However, in the second phase, O-alkylated (HCOH) carbons were gradually eliminated and fused-ring aromatic structures (128 ppm) became the predominant component. Meanwhile, the NMR bands strongly overlapped in the region of 50–100 ppm for biochars less than 300°C and in the region of 100–150 ppm for biochars more than 300°C. Similar results were observed by Zhang et al. [24], who investigated charred corn straw in the range of 100–600°C by ^13^C NMR spectroscopy and found that with increasing charring temperature, the trends of total aliphatic C (0–93 ppm) contents decreased, while the total aromatic C (93–165 ppm) contents increased. Rutherford et al. also demonstrated that the initial loss of material was attributable to aliphatic components, which were either lost or converted to aromatic carbon in the charring process [25]. Recently, Cao et al. also observed that the similar temperature-dependent ^13^C NMR spectroscopy of wood chars, suggesting that heat treatment at 300°C resulted in a material that was composed primarily of residues of biopolymers such as lignin and cellulose; carbohydrates were completely lost for char prepared at 350°C; at 400°C and above, the char lost ligno-cellulosic features and consisted predominantly of aromatic structures [23].

Because the functional groups in one-dimensional ^13^C NMR spectra strongly overlapped, no further analysis is possible from cursory observations of the original spectra. Therefore, in this study, 2D correlation analysis was applied to further investigate the development of functional groups with charring temperature.

Analysis of 2D ^13^C NMR correlation spectroscopy produces synchronous and asynchronous maps. The correlation peaks in the synchronous map are composed of autopeaks and crosspeaks, which appear at both diagonal and off-diagonal positions, respectively. Autopeaks represent the overall susceptibility of the corresponding spectral region to change in spectral intensity as an external perturbation is applied to the system, while crosspeaks represent simultaneous or coincidental changes of spectral intensities observed at two different spectral variables. An asynchronous spectrum has no autopeaks and consists exclusively of crosspeaks located at off-diagonal positions. This spectrum provides useful information on the sequential order of events observed by the spectroscopic technique along the external variable.

**Figure 3:**
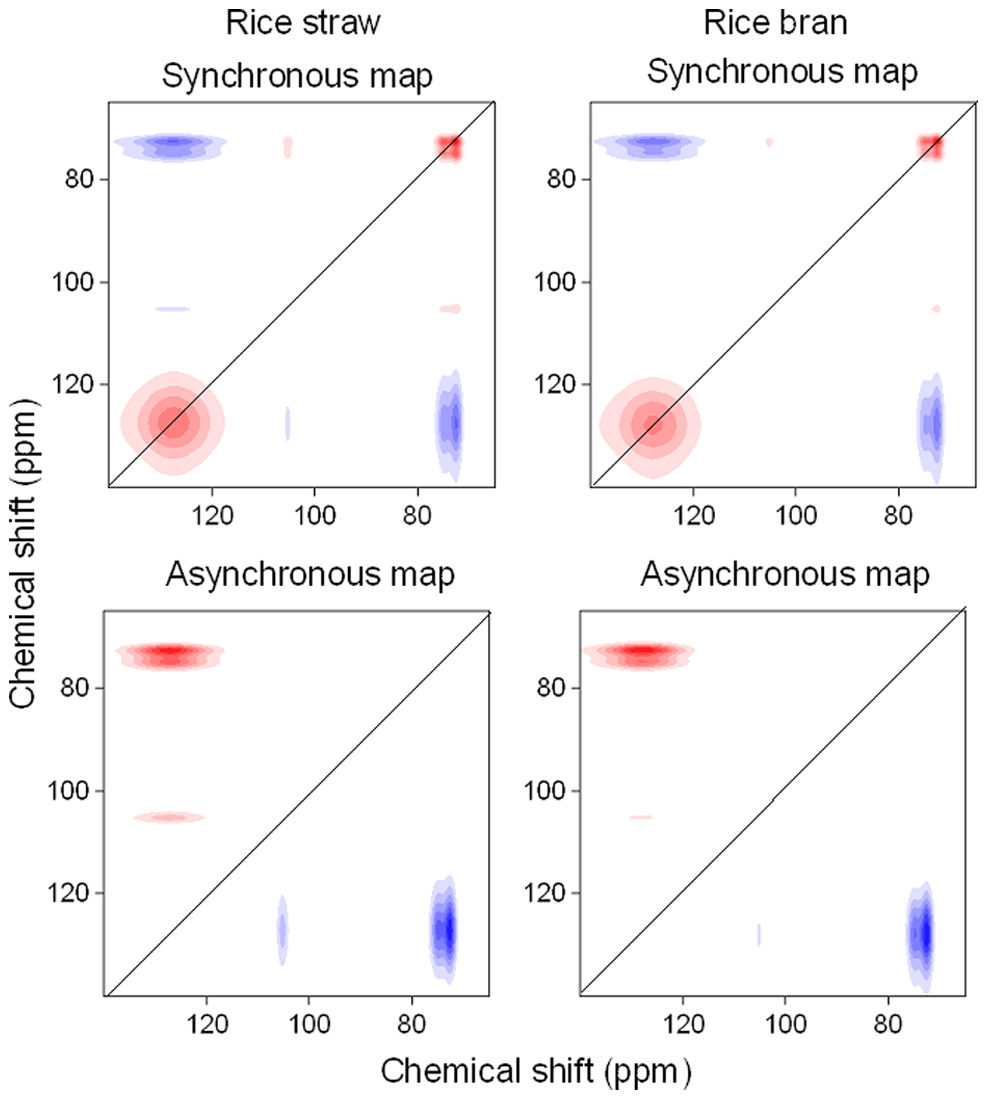
Synchronous and asynchronous 2D ^13^C NMR maps of rice straw and rice bran derived biochars over charring temperatures (100∼600°C). Red represents positive correlation, and blue represents negative correlation; a higher color intensity indicates a stronger positive or negative correlation.

The 2D ^13^C NMR correlation spectroscopy showed that for both biochars, the synchronous and asynchronous maps were similar (Figure 3). The synchronous maps displayed two major positive autopeaks at 128 ppm and 73 ppm, indicating that of all the functional groups, both fused-ring aromatic structures (128 ppm) and aliphatic O-alkylated (HCOH) (73 ppm) carbons intensity changed with increasing charring temperature. Therefore, the production of biochars is a process of dealkylation, dehydroxylation/dehydrogenation and aromatization of plant biopolymers, which was consistent with the results of the elemental analysis (Figure 1). It was noted from the synchronous maps that the highest change in intensity was in the band at 128 ppm, followed by 73 ppm, suggesting that with increasing charring temperature, fused-ring aromatic structures changed much more than aliphatic O-alkylated (HCOH) carbons. Moreover, one positive crosspeak at Φ(105, 73) and two negative crosspeaks at Φ(128, 73) and Φ(128, 105) were identified, reflecting that aliphatic O-alkylated (HCOH) carbons and anomeric O-C-O carbons at approximately 105 ppm co-vary with increasing charring temperature, whereas aliphatic O-alkylated (HCOH) carbons had an opposite variation trend with fused-ring aromatic structures. This result was consistent with the observation from the one-dimensional spectrum that aliphatic O-alkylated (HCOH) (i.e., 73 ppm) carbons decreased but fused-ring aromatic structures (i.e., 128 ppm) increased (Figure 2).

The asynchronous maps exhibited two major positive crosspeaks at Ψ(128, 73) and Ψ(128, 105) above the diagonal line for both biochars. Based on the Noda's rules [17], the sequence of the change of bands with increasing charring temperature followed the order 73 ppm (105 ppm)→128 ppm. Therefore, with increasing charring temperature, the mass cleavage of aliphatic O-alkylated (HCOH) groups and anomeric O-C-O carbons occurred prior to the production of fused-ring aromatic structures, which was consistent with the observation of one-dimensional ^13^C NMR spectra (Figure 2).

**Figure 4:**
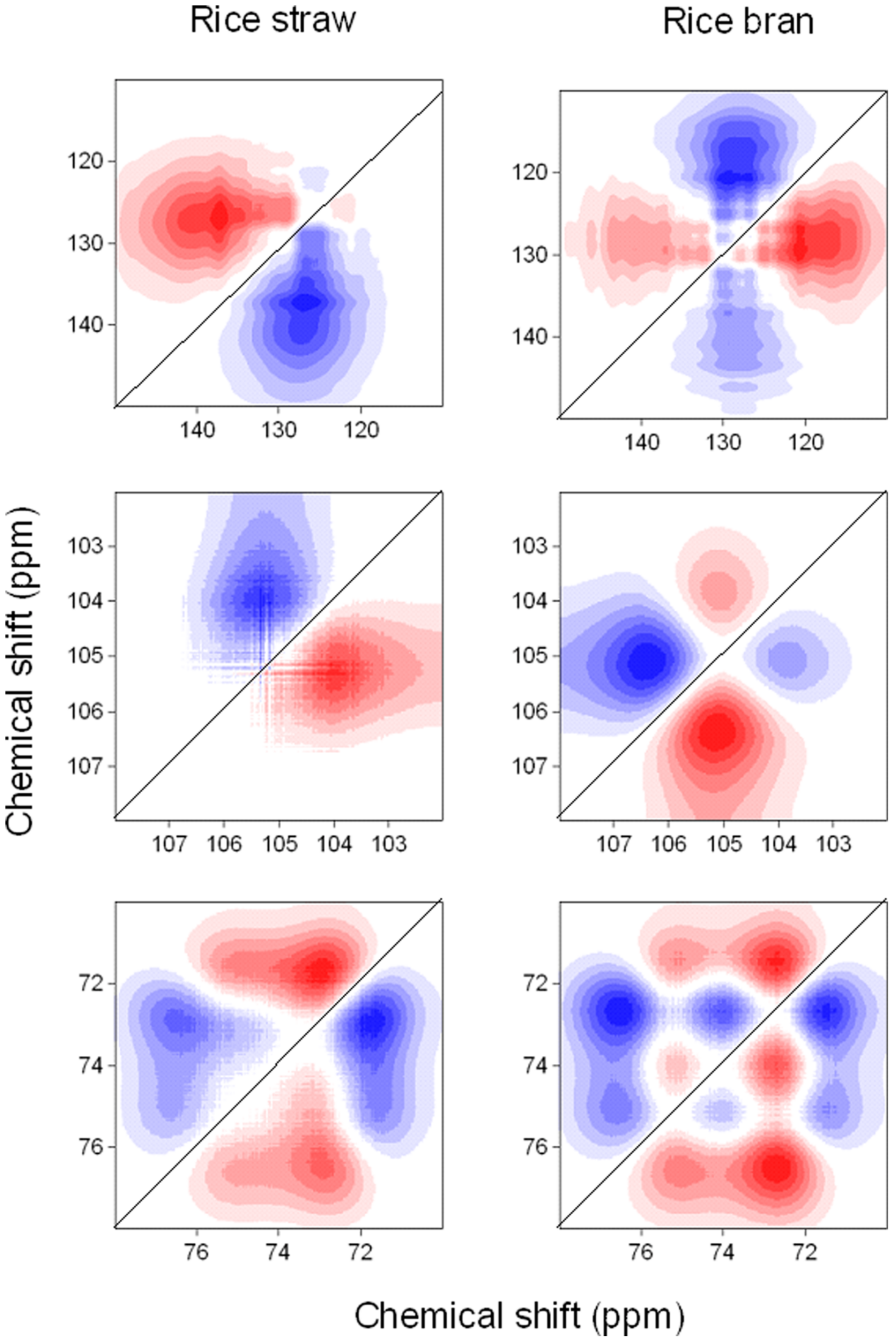
Asynchronous 2D ^13^C NMR maps of rice straw and rice bran derived biochars over charring temperatures (100∼600°C) in a shorten range of bands. Red represents positive correlation, and blue represents negative correlation; a higher color intensity indicates a stronger positive or negative correlation.

Although the asynchronous maps derived from the NMR region of 65–140 ppm were similar for both biochars (Figure 3), subtle differences could be found in the asynchronous map in the small NMR regions (Figure 4). Of these changes, none of the bands was common to both plant biomasses, reflecting that all of the changes in the functional groups were specific to the nature of the plant materials. The band at 127 ppm (Figure S1) was overlapped by 140 and 127 ppm for rice straw derived biochars, but by 140, 127 and 117 ppm for rice bran derived biochars. Only one major negative crosspeak located at Ψ(105, 104) was found in charred rice straw; however, both negative and positive crosspeaks located at Ψ(106.5, 105) and Ψ(105, 104) were observed in charred rice bran, showing that the band at 105 ppm presented in the synchronous map (Figure S1) was overlapped by 2 bands (i.e., 105 and 104 ppm) and 3 bands (i.e., 106.5, 105 and 104 ppm) for rice straw and rice bran derived biochars, respectively. Similarly, in the region of 78–70 ppm, 2 [i.e., Ψ(76.4, 73) and Ψ(73, 72)] and 6 [Ψ(76.4, 75.3), Ψ(76.4, 73), Ψ(75.3, 74), Ψ(75.3, 71.5), Ψ(74, 73) and Ψ(73, 71.5)] major crosspeaks were found in rice straw and rice bran derived biochars, respectively, demonstrating the band at 73 ppm (Figure S1) overlapping by 3 bands (76.4, 73 and 72 ppm) and 5 bands 76.4, 75.3, 74, 73, 72 and 71.5 ppm) for rice straw and rice bran derived biochars, respectively.

The sequencing of the change of overlapping bands with increasing charring temperature could also be identified based on Noda's rules [17]. Specifically, the production of aromatic C-O groups (140 ppm) occurred prior to the removal of fused-ring aromatic structures (127 ppm) for rice straw derived biochars; however, the removal of fused-ring aromatic structures (117 and 127 ppm) occurred prior to the production of aromatic C-O groups (140 ppm) for rice bran derived biochars. For anomeric O-C-O carbons the mass cleavage of the band at 104 ppm occurred prior to that of the band at 105 ppm for rice straw derived biochars; however, it follows the order: 105 ppm>104 ppm or 106.5 ppm for rice bran derived biochars. The mass cleavage of aliphatic O-alkylated (HCOH) groups follows the order: 73 ppm>72 ppm>76.4 ppm for rice straw derived biochars and 73 ppm>75.3 ppm>72 ppm>74 ppm>76.4 ppm for rice bran derived biochars. In summary, the asynchronous maps could provide the information about the overlapping bands and be used to identify the plant materials.

The regression analysis between functional groups and charring temperatures demonstrated that functional groups were linear with charring temperatures (Table S1). The slope of the regression equation suggested that the development rate of functional groups followed the order: fused-ring aromatic structures (127 and 128 ppm, slope≈0.03)>aromatic C-O groups (140 ppm, slope≈0.02)>aliphatic O-alkylated (HCOH) carbons (72, 73, 75.3 and 76.4 ppm, slope≈0.002)≈anomeric O-C-O carbons (104, 105 and 106.5 ppm, slope≈0.002). This result reveals that with increasing charring temperatures, the aromatization process is faster than the dealkylation and dehydroxylation/dehydrogenation processes. Meanwhile, one could design the “desired” biochars by choosing a suitable charring temperature.

In summary, 2D ^13^C NMR correlation spectroscopy demonstrated that the agricultural biomass carbonized to biochars consisted of dealkylation, dehydroxylation/dehydrogenation and aromatization processes, mainly involving the cleavage of aliphatic O-alkylated (HCOH) carbons and anomeric O-C-O carbons, as well as the production of fused-ring aromatic structures and aromatic C-O groups. When compared to one-dimensional ^13^C NMR spectroscopy, 2D ^13^C NMR correlation spectroscopy solves the problem of overlapping bands and enables researchers to probe the specific sequencing of spectral intensity changes of biochars.

### Establishment of the Relationship between Biochar Properties (pH and EC) and Functional Groups

The development of functional groups determined the pH and EC of biochars. Pearson correlation analysis was used to establish the relationship between biochar properties (pH and EC) and functional groups (Table 2). For rice straw derived biochars, the pH was negatively correlated (*R*>−0.82, *p*<0.05) with aliphatic O-alkylated (HCOH) carbons (72, 73 and 76.4 ppm) and anomeric O-C-O carbons (104 and 105 ppm), but positively correlated with fused-ring aromatic structures (120 and 128 ppm) (*R* = 0.99, *p*<0.05) and aromatic C-O groups (140 ppm) (*R* = 0.94, *p*<0.01) (Table 2). The EC of charring rice straw was also negatively correlated (*R*>−0.86, *p*<0.05) with aliphatic O-alkylated (HCOH) carbons (72, 73, and 76.4 ppm), but positively correlated with fused-ring aromatic structures (127 and 128 ppm) (*R* = 0.92, *p*<0.01) and aromatic C-O groups (140 ppm) (*R* = 0.89, *p*<0.05). For rice bran derived biochars, the pH was negatively correlated (*R*>−0.82, *p*<0.05) with aliphatic O-alkylated (HCOH) carbons (72, 73, 75.3 and 76.4 ppm) and anomeric O-C-O carbons (104, 105 and 106.5 ppm), but positively correlated with fused-ring aromatic structures (117, 127 and 128 ppm) (*R*>0.93, *p*<0.01) and aromatic C-O groups (140 ppm) (*R* = 0.95, *p*<0.01) (Table 2). However, the EC of charring rice bran had no correlation with any of the functional groups.

**Table 2:** Pearson correlation coefficiency (*R*) between properties (pH, EC) and functional groups derived from solid state ^13^C NMR spectroscopy.^a^

		NMR band (ppm)										
		72	73	75.3	76.4	104	105	106.5	117	127	128	140
pH	Rice straw	−0.87*	−0.87**	NA	−0.88*	−0.82*	−0.82*	NA	NA	0.99**	0.99**	0.94**
	Rice bran	−0.83*	−0.82*	−0.84*	−0.86*	−0.89*	−0.86*	−0.95**	0.93**	0.99**	0.99**	0.95**
EC	Rice straw	−0.87*	−0.86*	NA	−0.88*	−0.79	−0.79	NA	NA	0.92**	0.92**	0.89*
	Rice bran	−0.01	0.01	−0.02	−0.07	−0.14	−0.07	−0.30	0.24	0.52	0.52	0.33

a Note:

* *p*<0.05;

** *p*<0.01; EC, electrical conductivity; NA, not available.

The regression analysis between functional groups and biochar properties (pH and EC) (Table S2) further demonstrated that the pH and EC of rice straw derived biochars were mainly determined by fused-ring aromatic structures (127 and 128 ppm) and anomeric O-C-O carbons (104 and 105 ppm), which had a high slope when compared to other functional groups. However, the pH of rice bran derived biochars was determined by both fused-ring aromatic structures (127 and 128 ppm) and aliphatic O-alkylated (HCOH) carbons (72, 73 and 75.3 ppm) with a slope of 0.7.

### Significance of This Work

The application of biochar to soil will enhance soil fertility and carbon sequestration [26]. However, understanding the development of functional groups and the methods that are most appropriate to determining them has lagged [8]. This type of information is needed to optimize the properties of biochar for specific purposes, such as pH amelioration, nutrient retention, or sequestration of soil organic matter. In this study, two-dimensional correlation spectroscopy was employed to investigate the development of functional groups with increasing charring temperature. Two-dimensional correlation spectroscopy could solve the peak overlapping problem of biochars, which is helpful for establishing the relationship between biochar properties and functional groups. It is possible to “design” the desired biochars for a given application by applying 2D correlation spectroscopy. For example, one could produce biochars with a high pH and EC to apply them to acidic soils for pH amelioration, by controlling the high percentage of aromatic carbons (fused-ring aromatic structures and aromatic C-O groups). Moreover, 2D correlation spectroscopy could also give the sequencing of functional groups changes. One could produce the desired biochars with special functional groups based on the sequencing of functional groups changes. In summary, two-dimensional correlation spectroscopy provides novel insights into the development of functional groups in biochars.

## Supporting Information

Table S1 **Regression Analysis between Functional Groups (I/I_0_) with Charring Temperatures (T).**
(DOC)

Table S2 **Regression Analysis between Biochar Properties (pH and EC) and Functional Groups (I/I_0_) Derived from Solid State ^13^C NMR Spectroscopy.**
(DOC)

Figure S1 **Synchronous 2D ^13^C NMR maps of rice straw and sawdust derived biochars over charring temperatures (100∼600°C).** Red represents positive correlation, and blue represents negative correlation; a higher color intensity indicates a stronger positive or negative correlation.
(DOC)
