# Supplementary material for: Functional Groups Determine Biochar Properties (pH and EC) as Studied by Two-Dimensional 13C NMR Correlation Spectroscopy
Source: PLoS One. 2013 Jun 19;8(6):e65949. doi: 10.1371/journal.pone.0065949 (PMC3686859; doi:10.1371/journal.pone.0065949)
Supplement: Table S1 — Regression Analysis between Functional Groups (I/I0) with Charring Temperatures (T). (DOC) [file pone.0065949.s001.doc]

| **Table S1.** Regression analysis between functional groups (I/I_0_) with charring temperatures (T)*^a^* | | | | |
| --- | --- | --- | --- | --- |
| NMR band (ppm) | Parameters of regression equation | | | |
|  | Equation | *R*^2^ | *p* | *n* |
| 72 | I/I_0_=1.051-0.0022T | 0.79 | < 0.001 | 12 |
| 73 | I/I_0_=1.038-0.0021T | 0.77 | < 0.001 | 12 |
| 75.3 | I/I_0_=1.045-0.0022T | 0.78 | < 0.05 | 6 |
| 76.4 | I/I_0_=1.072-0.00223T | 0.83 | < 0.01 | 12 |
| 104 | I/I_0_=1.075-0.0018T | 0.76 | < 0.05 | 12 |
| 105 | I/I_0_=1.065-0.0019T | 0.74 | < 0.05 | 12 |
| 106.5 | I/I_0_=1.234-0.0020T | 0.91 | < 0.05 | 6 |
| 117 | I/I_0_=-0.257+0.0017T | 0.92 | < 0.05 | 6 |
| 127 | I/I_0_=-3.902+0.0340T | 0.96 | < 0.001 | 12 |
| 128 | I/I_0_=-4.255+0.0373T | 0.95 | < 0.001 | 12 |
| 140 | I/I_0_=-1.742+0.0299T | 0.83 | < 0.001 | 12 |
| *^a^*Note that I, I_0_ represent the intensity of NMR band (ppm) at temperature “T” and 100^o^C, respectively. | | | | |
