# Supplementary material for: Functional Groups Determine Biochar Properties (pH and EC) as Studied by Two-Dimensional 13C NMR Correlation Spectroscopy
Source: PLoS One. 2013 Jun 19;8(6):e65949. doi: 10.1371/journal.pone.0065949 (PMC3686859; doi:10.1371/journal.pone.0065949)
Supplement: Table S2 — Regression Analysis between Biochar Properties (pH and EC) and Functional Groups (I/I0) Derived from Solid State 13C NMR Spectroscopy. (DOC) [file pone.0065949.s002.doc]

| **Table S2.** Regression analysis between biochar properties (pH and EC) and functional groups (I/I_0_) derived from solid state ^13^C NMR spectroscopy *^a^* | | | | |
| --- | --- | --- | --- | --- |
| NMR band (ppm) | | Parameters of regression equation | | |
|  |  | Equation | *R*^2^ | *p* |
| Rice straw | 72 | pH=1.703-0.178 I/I_0_ | 0.70 | < 0.05 |
|  | 73 | pH =1.673-0.175 I/I_0_ | 0.68 | < 0.05 |
|  | 76.4 | pH =1.722-0.180 I/I_0_ | 0.72 | < 0.05 |
|  | 104 | pH =1.468-0.129 I/I_0_ | 0.58 | < 0.05 |
|  | 105 | pH =1.517-0.140 I/I_0_ | 0.59 | < 0.05 |
|  | 127 | pH =-14.07+2.75 I/I_0_ | 0.97 | < 0.001 |
|  | 128 | pH =-14.63+2.878 I/I_0_ | 0.97 | < 0.001 |
|  | 140 | pH =-7.60+1.921 I/I_0_ | 0.86 | < 0.001 |
|  | 72 | EC=2.369-0.351 I/I_0_ | 0.76 | < 0.05 |
|  | 73 | EC =2.336-0.346 I/I_0_ | 0.75 | < 0.05 |
|  | 76.4 | EC =2.392-0.354 I/I_0_ | 0.84 | < 0.05 |
|  | 104 | EC =1.910-0.248 I/I_0_ | 0.71 | > 0.05 |
|  | 105 | EC =2.000-0.270 I/I_0_ | 0.71 | > 0.05 |
|  | 127 | EC =-22.38+5.102 I/I_0_ | 0.97 | < 0.001 |
|  | 128 | EC =-23.28+5.320 I/I_0_ | 0.94 | < 0.001 |
|  | 140 | EC =-13.68+3.603 I/I_0_ | 0.76 | < 0.05 |
| Rice bran | 72 | pH=5.545-0.629 I/I_0_ | 0.61 | < 0.05 |
|  | 73 | pH =7.047-0.797 I/I_0_ | 0.59 | < 0.05 |
|  | 75.3 | pH =4.685-0.532 I/I_0_ | 0.63 | < 0.05 |
|  | 76.6 | pH =2.062-0.236 I/I_0_ | 0.68 | < 0.05 |
|  | 104 | pH =0.708-0.074 I/I_0_ | 0.75 | < 0.05 |
|  | 105 | pH =1.498-0.157 I/I_0_ | 0.68 | < 0.05 |
|  | 106.5 | pH =0.501-0.050 I/I_0_ | 0.87 | < 0.05 |
|  | 117 | pH =-0.418+0.097 I/I_0_ | 0.82 | < 0.05 |
|  | 127 | pH =-3.890+0.724 I/I_0_ | 0.97 | < 0.001 |
|  | 128 | pH =-3.998+0.744 I/I_0_ | 0.98 | < 0.001 |
|  | 140 | pH =-0.584+0.117 I/I_0_ | 0.87 | < 0.05 |
| *^a^*Note that I, I_0_ represent the intensity of NMR band (ppm) at temperature “T” and 100^o^C, respectively. | | | | |
