# Supplementary material for: Functional Groups Determine Biochar Properties (pH and EC) as Studied by Two-Dimensional 13C NMR Correlation Spectroscopy
Source: PLoS One. 2013 Jun 19;8(6):e65949. doi: 10.1371/journal.pone.0065949 (PMC3686859; doi:10.1371/journal.pone.0065949)
Supplement: Figure S1 — Synchronous 2D 13C NMR maps of rice straw and sawdust derived biochars over charring temperatures (100∼600°C). Red represents positive correlation, and blue represents negative correlation; a higher color intensity indicates a stronger positive or negative correlation. (DOC) [file pone.0065949.s003.doc]

**Figure S1. Synchronous 2D ^13^C NMR maps of rice straw and sawdust derived biochars over charring temperatures (100~600^o^C)**. Red represents positive correlation, and blue represents negative correlation; a higher color intensity indicates a stronger positive or negative correlation.
